# Identification of DOT1L inhibitor in a screen for factors that promote dopaminergic neuron survival

**DOI:** 10.3389/fnagi.2022.1026468

**Published:** 2022-12-12

**Authors:** Jun Cui, Joseph Carey, Renee A. Reijo Pera

**Affiliations:** ^1^Department of Cell Biology and Neuroscience, Montana State University, Bozeman, MT, United States; ^2^McLaughlin Research Institute, Great Falls, MT, United States

**Keywords:** Parkinson’s disease, DOT1L, DOT1L inhibition, SGC0946, dopaminergic neuron, high throughput screen, neural progenitor cells, human pluripotent stem cell

## Abstract

Parkinson’s disease (PD) is a common neurodegenerative disorder characterized by the progressive loss of dopaminergic (DA) neurons in the substantia nigra region of the midbrain. Diagnostic criteria for PD require that at least two of three motor signs are observed: tremor, rigidity, and/or bradykinesia. The most common and effective treatment for PD is Levodopa (L-DOPA) which is readily converted to DA and has been the primary treatment since the 1960’s. Dopamine agonists have also been developed but are less effective than L-DOPA. Although the lack of a model system to study PD has hampered efforts to identify treatments, diverse screening strategies have been proposed for identification of new pharmaceutical candidates. Here, we describe a pilot screen to identify candidate molecules from a bioactive compound library, that might increase formation, maintenance and/or survival of DA neurons *in vitro*. The screen used a previously characterized reporter construct consisting of the luciferase gene inserted downstream of the endogenous tyrosine hydroxylase (TH) gene and neurons differentiated from human pluripotent stem cells for 18 days. The reporter mimics expression of TH and includes a secreted luciferase whose activity can be measured non-invasively over multiple timepoints. Screening of the bioactive compound library resulted in the identification of a single molecule, SGC0946, that is an inhibitor of DOT1L (Disruptor Of Telomeric silencing 1-Like) which encodes a widely-conserved histone H3K79 methyltransferase that is able to both activate and repress gene transcription. Our results indicate that SGC0946 increased reporter luciferase activity with a single treatment for 48-h post-plating being equivalent to continuous treatment. Moreover, data suggested that the total number of neurons differentiated in the assays was comparable from experiment to experiment under different SGC0946 treatments over time. In contrast, data suggested that the survival and/or maintenance of DA neurons might be specifically enhanced by SGC0946 treatment. These results document the feasibility of a set of tools for further exploration of small molecules that may impact DA neuron differentiation, maintenance and/or survival. Results provide evidence in support of other reports that indicate inhibition of DOT1L may play an important role in maintenance and survival of neural progenitor cells (NPCs) and their lineage-specific differentiation.

## Introduction

Parkinson’s disease (PD) is a common neurodegenerative disorder that affects almost 1 million people in the United States and more than 6 million internationally (GBD 2016 Parkinson’s Disease Collaborators, 2018). Although the symptoms of PD vary among patients, a central hallmark is the progressive loss of movement control associated with the progressive loss of dopaminergic (DA) neurons of the substantia nigra of the midbrain (Albin et al., 1995). Intensive study over the years has identified numerous environmental and genetic risk factors that lead to PD in a subset of cases; however, the cause of the majority of PD remains poorly understood (Bloem et al., 2021).

One of the major limitations that hinders research in PD is the lack of access to affected human DA neurons. Thus, the development of human pluripotent cells including both human embryonic stem cells (hESCs) and induced pluripotent stem cells (iPSCs) has demonstrated the potential to provide a reliable source to obtain human DA neurons and study the disease. hESCs are pluripotent cells that have been derived from the inner cell mass of an early-stage embryo (Thomson et al., 1998) while iPSCs are generated from adult somatic cells, usually skin fibroblast cells, that are induced to pluripotency *via* a combination of four transcription factors (Takahashi and Yamanaka, 2006; Takahashi et al., 2007). Advances over the last decade or so, in defining differentiation conditions have made it possible to directly drive differentiation of human pluripotent cells to DA neurons and use these derived neurons to model PD in a dish (Yang et al., 2008; Chambers et al., 2009; Swistowski et al., 2010; Kriks et al., 2011; Gonzalez et al., 2013; Zhang et al., 2014; Singh et al., 2017; Barbuti et al., 2020; Kedariti et al., 2022; Stern et al., 2022). Multiple studies from different research laboratories have also shown that these derived neurons not only have cellular and biochemical characteristics very similar to the neurons developed in the human brain but also are functional when transplanted into animal models and thus, may have the potential to contribute to regenerative treatment options for the disease (Byers et al., 2011; Kriks et al., 2011; Doi et al., 2014; Grealish et al., 2014; Byers et al., 2015; Hoban et al., 2020; Behl et al., 2022). Thus, studies over the last decade plus have demonstrated that human pluripotent stem cells can provide a reliable source for generating DA neurons for *in vitro* disease modeling.

Developing treatments for PD traditionally has focused predominantly on relief of symptoms, as there is currently no effective mean(s) to restore DA neurons that are lost in PD patients. One potentially feasible option is to restore these neurons in patients *via* transplantation of *in vitro* derived DA neurons. Another approach is to screen for chemicals that have a protective effect directly on DA neurons. Pharmaceutical chemicals that may improve survival of DA neurons have multiple uses in PD related research and future applications: The immediate application of such chemicals is to enhance the production of a larger number of DA neurons more efficiently in *in vitro* derivation of DA neurons. Further, pharmaceutical chemicals would also provide a window into PD to enable mapping of biological function(s) of pathways that are compromised in PD. Finally, such chemicals might also slow or halt loss of DA neurons *in situ* during progression of PD.

As indicated above, DA neurons differentiated *in vitro* have the potential to provide high quality human midbrain DA neurons that are fully functional both in *in vitro* and *in vivo* assays and applications. Yet, it is clear that current differentiation methods are still time consuming, and the number of derived neurons is often limited greatly by variation in the differentiation efficiencies from one experimental replicate to another. Applications that use derived DA neurons either for *in vitro* cellular modeling or for future cell replacement therapies would benefit greatly from the improvement of the differentiation methods and/or development of methods that distinguish DA neuron survival in a complex cell mixture.

In order to screen for chemicals that might enhance DA differentiation and/or survival, we used a genetic reporter system that we previously constructed and demonstrated was able to monitor differentiation efficiency and compare DA survival under different conditions (Cui et al., 2016). We note here that the reporter contains an insertion of a luciferase reporter gene into the endogenous TH locus, the gene that encodes the enzyme governing the rate-limiting step in dopamine production (Cui et al., 2016). The luciferase reporter was shown to enable rapid non-invasive quantification of dopaminergic neurons in cell culture throughout the entire differentiation process (Cui et al., 2016). Moreover, luciferase was shown to be under the same endogenous regulation as the TH gene demonstrating that the cellular assay is effective in assessing neuron response to different cytotoxic chemicals and able to be scaled for high throughput applications suggesting feasibility of use as a quantitative cellular model for toxin evaluation and drug discovery. Here, we present results of a pilot screening study, based on a well-characterized, commercially available Tocris chemical library, that was aimed at validating that the reporter system paired with pluripotent stem cells is useful for identifying compounds that may improve the survival of DA neurons during direct differentiation *in vitro*, in adherence to culture plates and/or in post-differentiation survival.

## Materials and methods

### The Tocris library

We chose to screen the Tocriscreen 2.0 library for potential factors that would act to promote differentiation, adherence and/or survival of DA neurons. This library was obtained from Bio-Techne Corporation (Minneapolis, MN) in a 96-well format with each chemical dissolved in 10 mM DMSO with an indicated shelf-life of at least 6 months. The 1,280 compounds within the Tocriscreen 2.0 library cover such research areas as cancer, immunology, neuroscience and stem cells. Pathways include the G-protein coupled receptors (GPCRs), kinases, enzymes and enzyme-linked receptors, chemicals that target pathways of cell biology and ion channels. For screening, day 18 DA neuron progenitors were plated and cultured for 10 days with DMSO only (control) or treated with chemicals during the first 48 h (single treatment) or 8 days (continuous treatment). TH expression was detected using the luciferase assay and normalized to the treatment with DMSO only. Results were graphed with X-axis representing the reading of single chemical treatment and Y-axis representing continuous treatment for 8 days. Luciferase results were confirmed by Q-PCR of the luciferase and TH RNA.

### Directed dopaminergic differentiation and quantification of TH using luciferase assay

Pluripotent human stem cells (hESCs) were differentiated toward a dopaminergic neuron cell fate following the floor-plate induction method (Kriks et al., 2011; Zhang et al., 2014). Cell supernatants were collected every day for the first 20 days of neuronal differentiation and every other day afterwards to ensure that the protocol produced DA neurons. Luminescent reactions were performed by mixing the supernatants with 10× reaction buffer consist of 300 mM Tris–HCl pH 8.0, 40 mM NaCl, 1% Triton X-100 and 200 μM substrate Coelenterazine (NanoLight; Flagstaff, AZ). Luminescent signal was measured using a Synergy H1 Plate Reader (BioTek; Santa Clara, CA). Background signal level was measured using blank medium with reaction buffer and subtracted from experimental data.

### Drug screening assays

hESCs were differentiated toward dopaminergic neurons for 18 days as described above. One parameter that directly affected the yield of DA neurons in the final culture in our previous study was cell density (Cui et al., 2016). To identify the optimal cell density for DA neuron differentiation and test performance of the TH luciferase reporter assay, we replated differentiated neural cells at different densities and followed the DA neuron growth for 2 weeks. When cells were plated at densities higher than 400 K cells per well, restraint by the surface growth area greatly limited neuron attachment and growth resulting in massive cell detachment within several days. In contrast, fewer than 50 K cells per well led to very poor cell attachment and a low total number of DA neurons. Within the optimal range, each well produced 10^3^ to 10^4^ units of luminescence signal when the culture matured and signals showed a linear correlation with cell numbers on Day 40, suggesting the cell growth was not restricted during the culturing period. The growth test was also performed in the 384-well format and exhibited a similar result with optimal cell densities of 20–100 K cells per well. These results indicated that the reporter is suitable for used as luciferase based quantitative assays within the optimal range of cells per well. Given these results, on Day 18, neurons were dissociated by incubating with Accutase (Innovative Cell Technologies; San Diego, CA) for 5 min. Dissociated cells were counted and replated to 96-well plates coated with poly-L-ornithine/laminin/fibronectin at a density of 3 × 10^5^ cells/cm^2^ and allowed to recover for 1 week in neurobasal medium containing B-27 supplement, BDNF, GDNF, cAMP, TGFb, and ascorbic acid. Drug treatment was performed as previously described for neurotoxins (Grealish et al., 2014; Cui et al., 2016). Fleshly prepared Tocris solutions were diluted with the basal medium and added to each well to reach the desired final concentration.

Assays were done with multiple wells of 4 replicates for each condition. Whole supernatants of the same well were collected before drug treatment and 48 h post treatment for quantification using luciferase assay as described above. Viability of TH+ cells was calculated as the percentage of luminescent signal intensity post treatment compared to that before treatment of the same well.

Note that the screen was designed to explore feasibility and to test which one of two alternatives (single or continual application) is more efficient to induce neurons to differentiate to a dopaminergic fate and subsequent maintenance of the neuron population. We then used qPCR to identify the time of maximal induction to distinguish between the early neuron fate commitment stage and later stages of induction or maintenance of the general expression of the genes.

### RNA extraction and quantitative PCR analysis

Total RNA was extracted using RNeasy (Qiagen, Hilden, Germany). For first-strand cDNA synthesis, 2 μg of total RNA was reverse transcribed with oligo-dT primers and SuperScript III reverse transcriptase (Life Technologies, Carlsbad, CA). Quantitative PCR reactions were run using Power SYBR PCR mix (Life Technologies, Carlsbad, CA) and detected by ABI 7300 Real-Time PCR System. Primers used for PCR are listed in Table 1. Relative quantity for each sample and gene was normalized and calculated based on the ∆Ct values using GAPDH as the control.

**Table 1:** Primers used in this study.

Th11-12 Forward	TGGAGTTCGGGCTGTGTAAG
Th11-12 Reverse	AGCTCCTGAGCTTGTCCTTG
Th-Luc Forward	CAGCCCTACCAAGACCAGAC
Th-Luc Reverse	CTCCCAGATGGTGAACAGGT
TUBB3 Forward	GGCCTTTGGACATCTCTTCA
TUBB3 Reverse	ATACTCCTCACGCACCTTGC
GIRK2 Forward	CGCTGATCATTAGCCATGAA
GIRK2 Reverse	ATGGGGTGCTGGTCTCATAG
NURR1 Forward	GGCGAACCCTGACTATCAAA
NURR1 Reverse	CTGGGTTGGACCTGTATGCT
DAT Forward	TGAGCTCTTCACGCTCTTCA
DAT Reverse	CACCATAGAACCAGGCCACT
GAPDH Forward	AAGGTGAAGGTCGGAGTCAACG
GAPDH Reverse	TGGACTCCACGACGTACTCAGC

### Statistical analysis

Three biological replicates were included in the secondary analysis quantitative PCR. T-tests were used to test the null hypothesis that the difference in group means is zero and an alternate hypothesis that the difference in group means is different from zero.

## Results

In order to screen for chemicals that might enhance DA differentiation and/or survival, we used a genetic reporter system that we previously constructed and demonstrated was able to monitor differentiation efficiency and compare DA survival under different conditions (Cui et al., 2016). As noted above, the reporter contains an insertion of a luciferase reporter gene into the endogenous TH locus and has been shown to enable rapid non-invasive quantification of dopaminergic neurons in cell culture throughout the entire differentiation process (Cui et al., 2016). We differentiated the reporter-containing pluripotent hESCs, previously described (Cui et al., 2016) to midbrain DA neurons using an established floor-plate induction protocol with defined combinations of growth factors (Kriks et al., 2011; Zhang et al., 2014). The engineered cells had previously been shown to differentiate to TH+ DA neurons with similar robustness as the parental cell line in both a 96-well and 384-well format, with stable expression of TH observed starting from Day 11 of induction and continuing to increase throughout the following culturing, which correlated with the timing of midbrain DA neuron growth and maturation (Cui et al., 2016).

**Figure 1:**
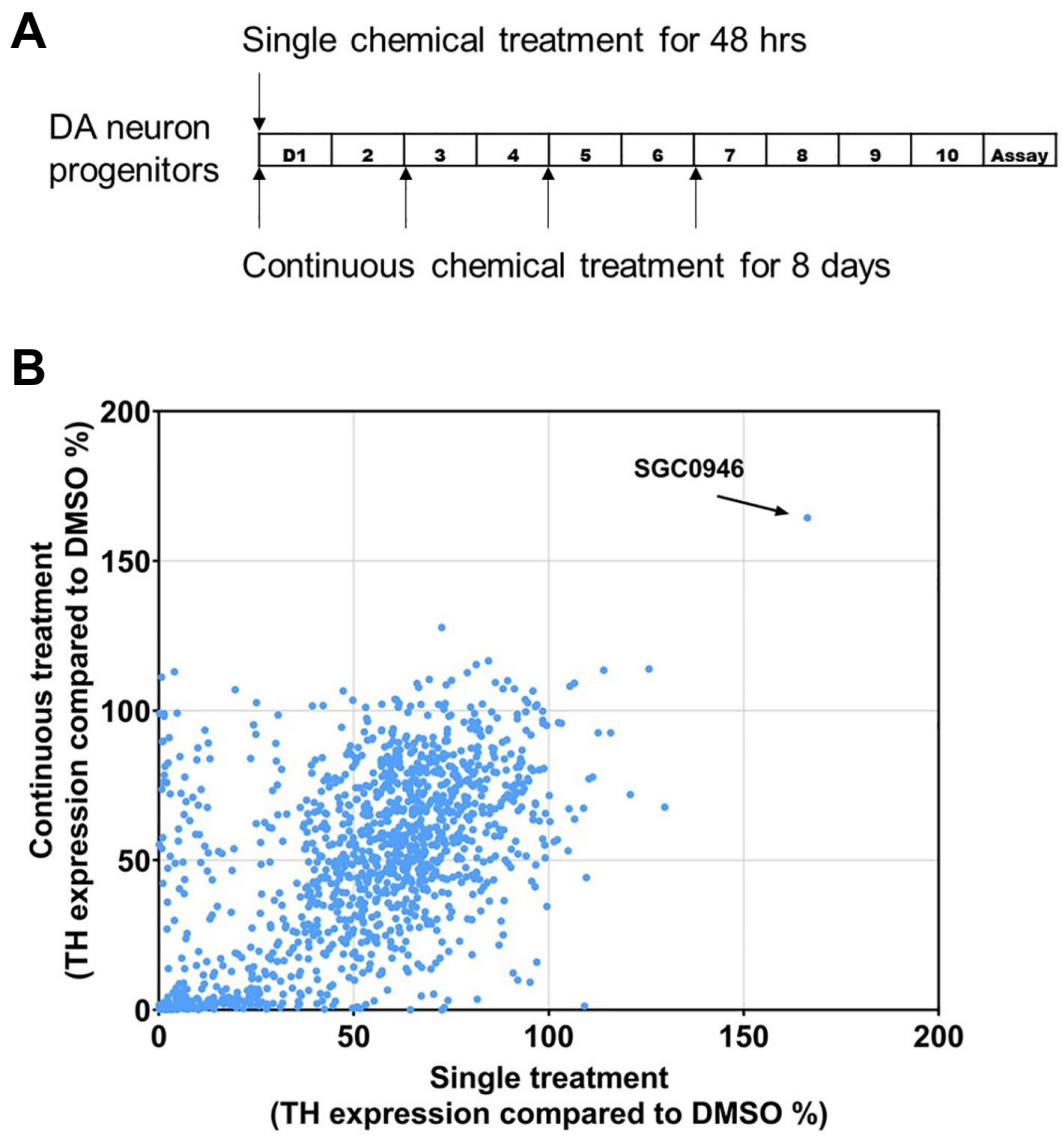
Screen of Tocriscreen 2 chemical library. **(A)** Experimental design. Day 18 DA neuron progenitors were plated and cultured for 10 days with DMSO only (Control) or treated with chemicals during the first 48 h (single treatment) or 8 days (continuous treatment). Luciferase readings were done at the end of the 10-day differentiation. **(B)** Screen results. TH expression was detected using the luciferase assay and normalized to the treatment with DMSO only. X-axis represents the reading of single chemical treatment and Y-axis represents continuous treatment for 8 days.

To screen the Tocriscreen 2.0 library, day 18 neural progenitors were plated and cultured for 10 days with DMSO only (control) or treated with chemicals during the first 48 h (single treatment) or 8 days (continuous treatment) as diagrammed (Figure 1A). TH expression was detected using the luciferase assay and normalized to the treatment with DMSO only. Results were graphed with X axis representing the reading of single chemical treatment and Y axis representing continuous treatment for 8 days. We chose to focus on graphing single chemical treatment vs. continuous treatment in order to identify factors that may promote the differentiation of day 18 neural progenitors to a dopaminergic fate.

We identified a single chemical component of the Tocriscreen 2.0 library, SGC0946, that resulted in the most significant increase (>165%) in reporter activity relative to the DMSO Only control (Figure 1B). Results suggested that one early pulse of SGC0946 is sufficient to induce neuron development and that potentially, the continuous treatment reinforces neuron maintenance and/or the lineage differentiation. We observed that multiple other chemical components of the Tocris library demonstrated smaller effects (increases up to 120% in reporter activity of single vs. continual treatments) while the majority of chemicals demonstrated a reduction in luciferase activity relative to the control (DMSO Only). Table 2 provides a list of the top 10 chemicals that demonstrated a positive response in the screen, along with a brief description of function.

**Table 2:** Top 10 positive responses to small molecules of the Tocriscreen 2.0 library^*^.

Single	Multiple	Average	Product name	Brief description
1.66338483	1.64401141	1.65369812	SGC 0946	Highly potent and selective DOT1L inhibitor
1.25677282	1.13910109	1.19793695	Lonidamine	Mitochondrial hexokinase inhibitor
1.14068884	1.13455792	1.13762338	RN 1734	Selective TRPV4 antagonist
1.06569007	1.0916159	1.07865298	SC 51089	Selective EP1 receptor antagonist
1.05430227	1.08110957	1.06770592	Ro 25–6,981 maleate	Subtype-selective NR2B antagonist
1.15839163	0.92565874	1.04202519	Sarpogrelate hydrochloride	Selective 5-HT2A antagonist
1.12654867	0.92557576	1.02606222	BD 1047 dihydrobromide	Sigma1 selective antagonist
0.95961761	1.06535556	1.01248658	DSR 6434	Potent TLR7 agonist
0.84524024	1.16597778	1.00560901	ML 193	Potent, selective GPR55 antagonist
0.72520622	1.27766667	1.00143644	GC 1	High-affinity thyroid receptor alpha (TRalpha) and TRbeta agonist

* The experimental design is as outlined in Methods and Figure 1. Day 18 DA neuron progenitors were plated and cultured for 10 days with DMSO only (Control) or treated with chemicals during the first 48 h (single treatment) or 8 days (continuous treatment). Luciferase readings were done at the end of the 10-day differentiation. Shown are the top 10 differential results in TH expression that was detected using the luciferase assay and normalized to the treatment with DMSO only when either a single treatment or continual treatment was employed.

**Figure 2:**
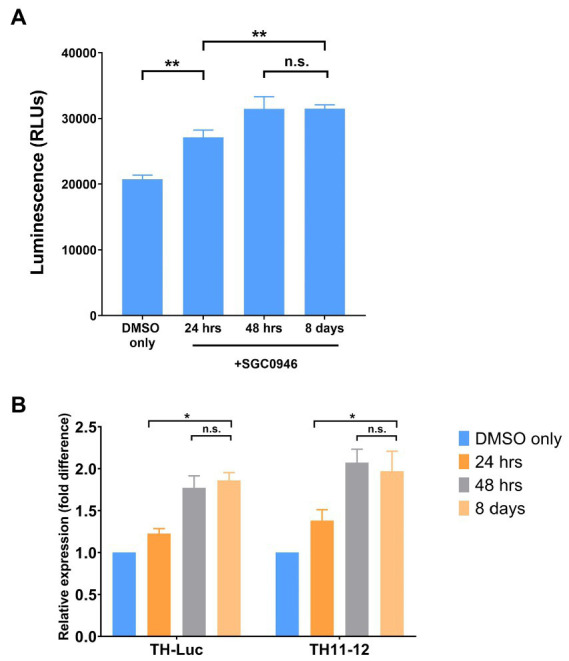
Tyrosine hydroxylase (TH) reporter activity. **(A)** TH expression by luciferase assay. Day 18 DA neuron progenitors were cultured for 10 days with DMSO only (Control) or treated with SGC0946 for the first 24 h, 48 h and 8 days. Luciferase readings were done at the end of the 10-day differentiation. **(B)** TH expression by qPCR of the luciferase gene (TH-Luc) or the endogenous TH gene (TH11-12). Experiments were performed in three replicate wells of neuron differentiation and drug treatment. Error bars indicate SEM. ^*^p < 0.05, ^**^p < 0.01, n.s., not significant.

We focused further on SGC0946 since it demonstrated the greatest increase in reporter activity and compared TH expression, as assessed by luciferase assay, to expression values obtained by qPCR of the luciferase gene insert (TH-Luc) or the endogenous TH gene (TH11-12). We observed that the luciferase readings of Day 18 plated DA neuron progenitors (following 10-days differentiation) corresponded to the TH expression by qPCR of both TH-Luc and TH11-12, as shown (Figures 2A,B).

**Figure 3:**
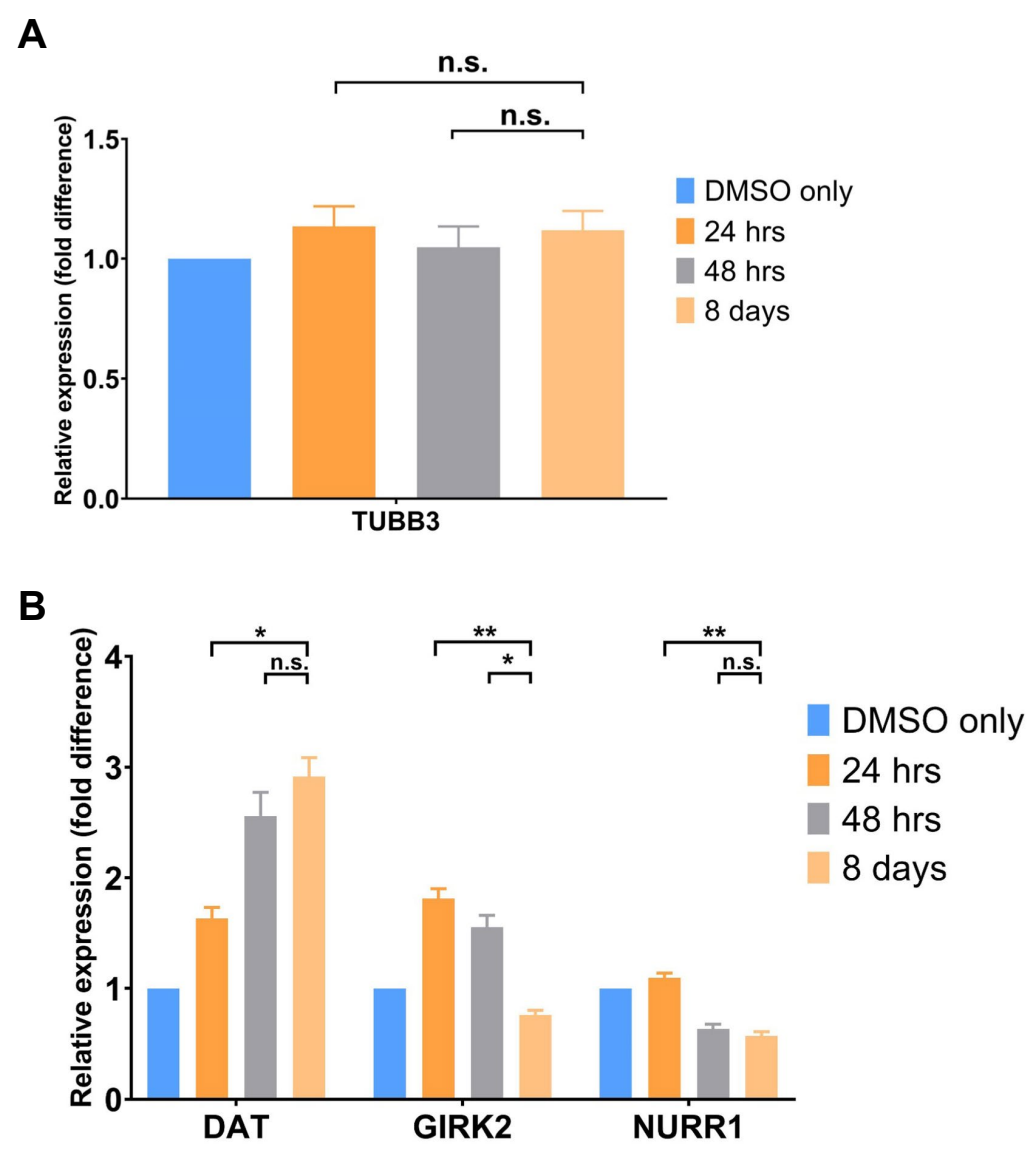
Gene Expression by qPCR. **(A)** SGC0946 treatment does not increase the number of total neurons as measured by expression of TUBB3 (BetaIII Tubulin). **(B)** SGC0946 treatment potentially increases the number of dopaminergic neurons as measured by the expression of TH and Dopamine Transporter (DAT). Data suggests that a short treatment is preferred for the midbrain DA neurons as measured by the midbrain DA neuron marker GIRK 2. NURR1 is a widely expressed marker on neurons, microglia and other cell types. Experiments were performed in three replicate wells of neuron differentiation and drug treatment. Error bars indicate SEM. ^*^p < 0.05, ^**^p < 0.01, n.s., not significant.

Finally, we compared gene expression of a marker of total neurons [TUBB3 (BetaIII Tubulin)] to markers that are reported to be specific to DA neurons [Dopamine Transporter (DAT), GIRK 2 and NURR1]. As shown in Figure 3A, there is no difference between control and treated total neurons following either single or continuous treatment with SGC0946. In contrast, markers specific to DA neurons (DAT, GIRK2 and to a lesser extent, NURR1) demonstrated an increase in expression with treatment with SGC0946 relative to DMSO Only control as shown (Figure 3B). A longer treatment of SGC0946 increases the expression of DAT, while the expression of GIRK2 and NURR1 can only benefit from a shorter period of SGC0946 treatment during the first 24 or 48 h of DA neuron differentiation.

## Discussion

Here, we report the use of a human pluripotent stem cell-based system, for discovery of neural toxins and protective factors, in a pilot screen for factors that might enhance differentiation, survival and/or maintenance of DA neurons differentiated *in vitro*. In this pilot screen, we identified SGC0946, an inhibitor of DOT1L (Disruptor Of Telomeric silencing 1-Like) which encodes a widely-conserved histone H3K79 methyltransferase that is able to both activate and repress gene transcription. Our results indicate that SGC0946 increased reporter luciferase activity with a single treatment at 8-h post-plating being equivalent to continuous treatment. Moreover, data indicated that the total number of neurons differentiated in the assays was comparable from experiment to experiment under different SGC0946 treatments over time.

Over the last decade or more, diverse screening strategies have been proposed for identification of new pharmaceutical candidates to treat PD. Strategies include high-content screening to generate single-cell gene-corrected patient-derived pluripotent stem cell clones useful for further exploration of excess alpha-synuclein with familial PD mutations and identification of perturbed pathways, two-step screening method to identify inhibitors of α-synuclein aggregation, high throughput screens of mitochondrial, neuron or neurite morphology, RPPA (Reverse Phase Protein Arrays), antibody-based proteomic approaches, and strategies that employ organoids, model organisms and virtual (*in silico*) screening and specific strategies such as the assembly of a “toolkit” to identify modulators of PARK7 (Lavecchia and Giovanni, 2013; Stewart, 2014; Smith et al., 2017; d’Amora and Giordani, 2018; Dawson et al., 2019; Barbuti et al., 2020; Aldewachi et al., 2021; Leah et al., 2021; Schikora et al., 2021; Hideshima et al., 2022).

Each of the compound screening strategies has strengths and limitations. For example, in Barbuti et al., the researchers used FACS analysis and high content screening to identify clonal iPSCs that were gene edited to correct SNCA (alpha synuclein gene) dosage; although not technically a drug screen, the methodology of using SNCA levels and FACS are applicable to drug screening (Barbuti et al., 2020). In a complementary approach, Hideshima et al. (2022) used a two-step screening with the thioflavin T assay and a cell-based assay to identify α-synuclein aggregation inhibitors *via* fluorescence monitoring. Casalino et al. (2022) used a novel approach with an luciferase reporter activity and a destabilized alpha-synuclein construct and demonstrated that the resultant destabilized fusion protein reporter system accurately reported a-synuclein levels in response to translation inhibitors and targeted siRNAs (Caslino et al., 2022). Subsequently they demonstrated the utility of this reporter in screening a focused library of 3,192 compounds. Other investigations focused on morphological changes and imaging to detect beneficial and/or detrimental pharmaceuticals. Finally, Jia et al. (2022) have assembled a “toolkit” that is promising for exploring PARK7 function in a physiologically relevant setting and for identifying and/or developing new and improved PARK7 inhibitors. The limitations of this collection of assays are numerous but often overcome with secondary screens; for example, the use of imaging, fluorescence and morphological alterations are susceptible to the natural dynamics that occur in cultures, under different fluorescent conditions and in non-specific (or off-target) pharmaceutical responses. Nonetheless, given the array of assays that are available for high throughput screening, it is hoped that the use of multiple assays might lead to positive intersections of the most promising treatments for PD, understanding of underlying causes and identification of growth factors and pathways that might be modulated.

As in other screens, there is a need to consider the limitations of this luciferase-based reporter screen. The first limitation that we noted was that many more chemicals appeared to have negative impacts, rather than positive impacts, on the differentiation, survival and/or maintenance of neural progenitors and differentiated dopaminergic neurons. Thus, negative modulation far outweighed positive. This requires that many more chemicals be screened to identify positive modulators. A second limitation of the pilot system that we outline here is the possibility that multiple methods may increase TH (and TH reporter) activity including the alteration of construct promoter activity directly or indirectly, stabilization of secreted luciferase protein, destruction of alternative pathways in differentiation (rather than promoting dopaminergic neuron differentiation, survival and/or maintenance). We previously characterized the TH reporter in this system (Cui et al., 2016) and demonstrated that the TH reporter activity reflected DA neuron quantity as demonstrated in experiments parallel to these in which differentiation of neurons was promoted. In addition, previous studies also demonstrated that TH expression was correlated with TH reporter activity, that dilution of DA neurons resulted in a reduction of the TH reporter activity and that the TH reporter reliably reported responses to neurotoxins (Cui et al., 2016). Thus, although the possibility of altered promoter activity in the screen cannot be discounted, further screening and secondary analysis will enable elucidation of the mechanisms underlying increased luciferase activity. In particular, measurements of dopamine content, in the presence of the drugs, as well as dopaminergic neuron activity with drug treatment would resolve the mode of action of the drug(s) identified in this pilot screen.

We note that a concern may exist regarding the relationship of DA neurons generated *in vitro* from pluripotent stem cells relative to neurons from the midbrain. Generally, the protocol used to differentiate neurons *in vitro* is a modified protocol originally proposed by Studer and colleagues and modified to enable generation of more mature DA neurons (Chambers et al., 2009; Byers et al., 2011; Kriks et al., 2011; Nguyen et al., 2011; Miller et al., 2013; Zhang et al., 2014; Kim Tw et al., 2021). The efficiency of DA neuron induction *in vitro* with these modified protocols has been shown to be similar across different cell lines of different genetic backgrounds including those carrying mutations in genes associated with PD such as the *SNCA* triplication (α-synuclein triplication) cell liner. When iPSC-derived DA neurons from PD-affected individuals are probed further, independent of genetic cause, they have been shown to display cardinal features of neurodegeneration including formation of α-synuclein aggregates, dysregulation of neurotransmission, and increased susceptibility of DA neurons, relative to non-DA neurons, to diverse neurotoxins as indicated by cell death and overexpression of markers of oxidative stress, culminating in cell death (Byers et al., 2011; Nguyen et al., 2011; Baena-Montes et al., 2021; Diao et al., 2021; Fukusumi et al., 2021; Lin et al., 2021; Drouin-Ouellet et al., 2022).

Whether the PD features observed correspond to changes *in vivo* in the midbrain was also addressed, at least in part, in studies that compared transcriptional profiles of human induced and primary midbrain dopaminergic neurons (Xia et al., 2016). These studies demonstrated that the transcriptomes of DA neurons derived from 6 different human pluripotent stem cell (hPSC) lines (from 3 control and 3 PD-affected individuals with *LRRK2, GBA* and *SNCA* mutations) and those of primary midbrain (mDA) neurons demonstrated distinct differences, particularly in terms of maturity of the neurons *in vitro*.(Xia et al., 2016) Nonetheless, one of the findings of this work was that although DA neurons are less mature than mDA neurons, a small subset of genes with altered expression in DA neurons from patients with PD demonstrated the same corresponding gene differences in *bona fide* PD human brain midbrain DA neurons. Thus, although there were differences between DA neurons generated *in vitro* from iPSCs and mDA neurons isolated from the brain in terms of global gene expression of neuron maturation genes, our data indicated that the transcriptomes of DA neurons from PD iPSCs reflect lesions of PD *in vivo* and are likely to be useful for *in vitro* PD modeling and chemical screening as outlined here (Xia et al., 2016). Moreover, recent studies of single cell transcriptomics further verified that although cell-to-cell variability occurs, there is robust expression of PD GWAS genes and overlap with postmortem adult substantia nigra neurons and that stress signatures were ameliorated with felodipine, an FDA-approved drug (Fernandes et al., 2020).

Together, the data reported here, in light of the characteristics of the *in vitro* differentiation system, suggest that the differentiation, survival and/or maintenance of DA neurons might be specifically enhanced by SGC0946 treatment. The results also suggest that perhaps one early pulse of the SGC0946 compound is sufficient to induce the neural phenotype, whereas the continuous treatment may reinforce the lineage differentiation. From the qPCR data, we also found that the maximal expression level of TH itself is observed in the first 48 h of treatment; the expression levels increased from 24 to 48 h but not significantly over 8 days. Thus, the early stage (the first 48 h) may represent the neuronal cell fate commitment stage and the drug may affect the fate determination rather than just inducing general expression of the genes. In addition, if we consider that the SGC0946 compound is an epigenetic remodeling reagent, the early induction suggests that the action of epigenetic modification controlled by the drug may be to sustain and/or direct the cell fate. Taken together, these results provide further evidence in support of other reports that indicate inhibition of DOT1L may play an important role in maintenance and survival of neural progenitor cells (NPCs) and their lineage-specific differentiation (Ferrari et al., 2020; Gray de Cristoforis et al., 2020).

Previously we performed a ChIP-sequencing study and characterized dopamine neuron transcriptional and epigenetic programs including the global binding profiles of H3K27ac, H3K4me1, and (5-hydroxymethylcytosine [5hmC]) at four different stages of development (Xia et al., 2017). We demonstrated the dynamic pattern of epigenetic modification during DA neuron differentiation and maturation. The discovery of the linkage between DOT1L and DA neurons in our compound screen here suggests that H3K79me might also play a key regulatory role during DA development.

As also noted in other publications and in a comprehensive review, DOT1-Like (DOT1L) is the sole methyltransferase of histone H3K79 (Wong et al., 2015; Ferrari et al., 2020; Gray de Cristoforis et al., 2020; Wille and Sridharan, 2022). DOT1L can orchestrate mono-, di-, or tri-methylation that is correlated with actively transcribing genes (mono-and di-methylation) or repression (tri-methylation). Moreover, DOT1L has been recognized as a promising epigenetic target for solid tumors and has been extensively studied in MLL (multi-lineage leukemia) genomic rearrangements and sporadic fusion proteins that may drive errors in the localization of H3K79 methylation and subsequent oncogenesis (Alexandrova et al., 2022). More recently, studies have demonstrated that loss of function of DOT1L is accompanied by perturbations in development of somatic lineages and in reprogramming *in vitro* (Wille and Sridharan, 2022). Thus, the evidence suggests that DOT1L is broadly required for differentiation, that reduced DOT1L activity is concomitant with increased developmental potential and that loss of DOT1L activity results in more upregulated than downregulated genes. DOT1L also participates in various epigenetic networks that are both cell type and developmental stage specific and may play an important role in maintenance and survival of neural progenitor cells (NPCs) and their lineage-specific differentiation (Ferrari et al., 2020; Gray de Cristoforis et al., 2020).

The precise role of DOT1L and histone H3K79 methylation in DA neurons or in PD is not known. Previous studies have examined the epigenome of DA neurons by reduced representation hydroxymethylation profiling (RRHP) sequencing on neurons differentiated *in vitro* and demonstrated that the distribution of 5hmC (5 hydroxymethyl cytosine) was associated with gene bodies (~85% of signals). Moreover, genes commonly implicated in DA neuron functions such as *TH* and *NR4A2* (which encodes NURR1, a differentiation and survival factor) are marked with 5hmC, as are more than 1,628 enhancers in *in vitro-*derived TH+ neurons. Notably in these studies, it was also observed that genes marked by 5hmC alone or by both 5hmC and histone modifiers have much higher expression levels compared to genes that are not marked by 5hmC with or without histone modifiers. This suggests that enhancer and 5hmC associated genes are indeed actively transcribing genes in TH+ DA neurons *in vitro*. Moreover, the highest intensity distribution was on cell type-specific gene, an observation in line with previous discoveries that cell specific genes have more 5hmC marks in ESCs and human brain samples (Szulwach et al., 2011; Wu et al., 2011; Khare et al., 2012).

In summary, it is hoped that this study may provide preliminary data with tools that may be useful to others in the community. Much remains to be learned about the relationship of DOT1L and DA neuron biology. Nonetheless, to our knowledge, this is the first association of DOT1L activity and formation, differentiation and/or maintenance of DA neurons. Clearly, further exploration of this association is merited.

## Data availability statement

The raw data supporting the conclusions of this article will be made available by the authors, without undue reservation.

## Author contributions

JCu, JCa, and RR contributed to the research described in this manuscript, experimental design, analysis of data, and interpretation of the results. JCu prepared the manuscript figures. JCa outlined the methods and materials section. RR prepared the final manuscript for publication. All authors contributed to the article and approved the submitted version.

## Funding

This work was supported by private funds from the Mallett Family (San Francisco, CA) and the Weissman family (Palo Alto, CA). The funders were not involved in the study design, collection, analysis, interpretation of data, the writing of this article or the decision to submit it for publication. McLaughlin Research Institute also supported this work.

## Conflict of interest

The authors declare that the research was conducted in the absence of any commercial or financial relationships that could be construed as a potential conflict of interest.

## Publisher’s note

All claims expressed in this article are solely those of the authors and do not necessarily represent those of their affiliated organizations, or those of the publisher, the editors and the reviewers. Any product that may be evaluated in this article, or claim that may be made by its manufacturer, is not guaranteed or endorsed by the publisher.
